# The evaluability bias in charitable giving: Saving administration costs or saving lives?

**Published:** 2014-07-01

**Authors:** Lucius Caviola, Nadira Faulmüller, Jim. A. C. Everett, Julian Savulescu, Guy Kahane

**Affiliations:** * Department of Experimental Psychology, University of Oxford, 9 South Parks Road, Oxford, OX1 3UD, U.K.; †Department of Experimental Psychology, University of Oxford; ‡ Oxford Uehiro Centre for Practical Ethics, University of Oxford; § Oxford Martin School, University of Oxford

**Keywords:** evaluability bias, overhead ratio, cost-effectiveness, charitable giving, pro-social behavior, altruism, cognitive bias

## Abstract

We describe the “evaluability bias”: the tendency to weight the importance of an attribute in proportion to its ease of evaluation. We propose that the evaluability bias influences decision making in the context of charitable giving: people tend to have a strong preference for charities with low overhead ratios (lower administrative expenses) but not for charities with high cost-effectiveness (greater number of saved lives per dollar), because the former attribute is easier to evaluate than the latter. In line with this hypothesis, we report the results of four studies showing that, when presented with a single charity, people are willing to donate more to a charity with low overhead ratio, regardless of cost-effectiveness. However, when people are presented with two charities simultaneously—thereby enabling comparative evaluation—they base their donation behavior on cost-effectiveness (Study 1). This suggests that people primarily value cost-effectiveness but manifest the evaluability bias in cases where they find it difficult to evaluate. However, people seem also to value a low overhead ratio for its own sake (Study 2). The evaluability bias effect applies to charities of different domains (Study 3). We also show that overhead ratio is easier to evaluate when its presentation format is a ratio, suggesting an inherent reference point that allows meaningful interpretation (Study 4).

## 1 Introduction

Concern about cost-effectiveness is prevalent in everyday life. When making a business decision, individuals want to know how much reward they will receive per dollar invested: “how much bang do I get for my buck?” At the same time, such thinking seems to be far less common in the context of altruistic decisions—or more specifically, donation to charities (Baron & Szymanska, 2011). When donating money with the intention of helping people in need, however, it is crucial to choose a charity based on its cost-effectiveness: how effective is that charity at doing good? (Ord, 2012). The question of charities’ cost-effectiveness is addressed by organizations conducting independent and scientific charity evaluation, such as *GiveWell*^1^ or *Giving What We Can*.^2^ These organizations have shown that some charities are more cost-effective in saving lives than others by a factor of up to one thousand (Jamison, 2006). Indeed, the lack of adequate prioritization and people’s inability to focus on charities’ cost-effectiveness probably leads to the death of many thousands of people who could have been saved, had the money been donated to more effective charities instead.

If the aim of charitable donation is to maximize the impact of one’s donation (a broadly consequentialist goal), then people should prefer to donate to those charities that are most cost-effective (Ord, 2012; Singer, 1979). Instead, however, people often donate more to charities with lower overhead ratios (i.e. administrative expenses), irrespective of their cost-effectiveness (Baron & Szymanska, 2011). A large-scale study revealed that over a third of US citizens believe both that a typical charity spends more than half of all donations on administration, and that charities *should* invest much less in administration (Sellers, 2012). As a result, charities are forced to keep overheads small in order fulfill the donors’ expectations (Goggins Gregory & Howard, 2009) and attempt to promote themselves by highlighting their low overhead ratio—with some charity evaluators even praising charities with low overhead ratios instead of focusing primarily on cost-effectiveness (Steinberg, 2010). For example, the organization *CARE* prominently advertises itself in the following way: “More than 90 percent of our expended resources—among the highest of all philanthropic organizations—support our poverty-fighting projects around the world. Less than 10 percent of expended resources go toward administrative and fund-raising costs.” (CARE, 2014).

The overhead ratio might appear important to many people because it can seem to measure the “efficiency” of the organization: how much of my money will actually reach the destination? This line of thinking would make sense in cases where the money that reached the destination had the same impact across charities. Often neglected, however, is the fact that some charities use more effective interventions than others and therefore have a much higher impact even though less money reaches the destination. In fact, studies have failed to find any correlation between overhead ratio and cost-effectiveness: organizations with a low overhead ratio can still achieve very little and organizations with a high overhead ratio can be very effective—perhaps because they are led by a good administration including competent staff, good infrastructure and self-evaluation (Bedsworth, 2008; Wing & Hager, 2004).

Interestingly, what seems to be a dominant attitude in the charity context sounds absurd when applied to the for-profit sector. Imagine a car company advertising its low overhead ratio: “90c of your dollar goes directly to building cars. Only 10% of our expenses go into planning, designing, [and advertising] them.” (Karnofsky, 2007).

It is not entirely clear yet why people focus on overhead ratio in the charity context. For example, when buying a car, people just want a maximum return for their money, even if that involves a more expensive overhead. It has been suggested by Baron and Szymanska (2011) that people focus on overhead ratio because it is easier to evaluate compared to cost-effectiveness. Their study has shown that people donate more to charities with lower overhead ratios, when they are presented with two equally cost-effective charities that differ in their overhead ratios. The proposal that people focus on overhead ratio due to its high evaluability (i.e., ease of evaluation) seems plausible but has not been explicitly tested in Baron and Szymanska’s nor in other studies. Presumably, as the overhead ratio is more readily available for most charities because it is straightforward to calculate, it becomes easier to choose a charity based on this aspect. Measuring cost-effectiveness, on the other hand, can be extremely difficult and requires extensive empirical research, as well as cognitive resources. In addition, ratios report the relationship of two numbers of the same scale (e.g., $200 out of $1,000 donated go into administration, i.e., 20%) and therefore imply a relative reference point for assessment. The open-ended scale of cost-effectiveness (e.g., saving 2 lives per $1,000 donated) is harder to evaluate. To the extent that the ease of evaluation can bias the weight given to an attribute, we may expect overhead ratio to appear more important than cost-effectiveness. In this paper we aim to investigate this question and the role of the *evaluability bias* in the context of charitable giving.

## 2 The psychology of the evaluability bias

We define the evaluability bias as *the tendency to weight the importance of an attribute in proportion to its ease of evaluation*, rather than based on criteria that are deemed as more relevant after reflection. To our knowledge, the evaluability bias has not been explicitly defined and discussed in the literature. However, many findings seem to support its existence. Prior research has identified a range of contexts in which people make judgments that are inconsistent with their values in cases where the evaluation of different options is involved (Hsee, 1996, 1998; Hsee & Zhang, 2010; Slovic, Finucane, Peters, & MacGregor, 2002). One prime example is scope insensitivity, the tendency to assign inappropriately low weight to the quantity or scope of the option in question (Kahneman, 2000). Hsee and Zhang (2010) assume that people often lack the knowledge to evaluate an option presented to them in isolation (i.e., how much weight to assign to this option) (general evaluability theory). However, if people are presented with multiple options simultaneously, i.e., in joint-evaluation, they have a reference point available, which enables them to compare different options. This increases the relative evaluability of the decision relevant attributes of these options. In cases where an option has both a difficult- and an easy-to-evaluate attribute, people mainly focus on the easy-to-evaluate attribute (Bazerman, Loewenstein, & White, 1992; Hsee, 1996). This literature has already pointed to the existence of the evaluability heuristic, the phenomenon that people’s judgments can be influenced by an option’s ease of evaluation.

We believe that the evaluability heuristic can lead to systematic suboptimal outcomes in many situations and thus refer to it as a cognitive bias. A cognitive bias is a systematic deviation from the normative model of rationality (Baron, 2005), which prevents people from effectively achieving their goals (e.g., in the context of charitable giving: helping as many people as possible per donation). We define the evaluability bias as the tendency to weight the importance of an attribute in proportion to its ease of evaluation, rather than based on criteria that are deemed as more relevant after reflection. We further assume that the evaluability bias is a general bias found in many people and that there is no bias going into the other direction, i.e., the tendency to overweight the importance of an attribute due to its low evaluability.

An empirical test of the evaluability bias is to observe whether people will shift their preferences in favor of a difficult-to-evaluate attribute, such as cost-effectiveness, when its evaluability is increased. If so, this would show that people tend to give disproportionate weight to an attribute just because of its greater evaluability. In a study by Hsee (1996) subjects were asked how much they would be willing to pay for a music dictionary. They were either presented with only one dictionary or with two dictionaries side-by-side. While dictionary A featured 10,000 entries and was in a good condition, dictionary B featured 20,000 entries and had a torn cover. Subjects who saw only one dictionary were willing to pay more for dictionary A, while subjects who saw both dictionaries were willing to pay more for dictionary B. Hsee assumes that people primarily prefer a dictionary with many entries but find it difficult to evaluate a dictionary on this dimension in separate-evaluation, because they do not know what qualifies as “many entries”. Instead, people focus on the condition of the dictionary (damaged vs. not damaged), which is easier to evaluate in separate-evaluation. In joint-evaluation, however, the number of entries becomes easier to evaluate resulting in the observed preference reversal. Thus, in cases where options are evaluated separately people mainly focus on attributes that are easy to evaluate, but in joint-evaluation people might focus on a different attribute because its evaluability is now higher.

Kogut and Ritov (2005) report a similar effect. When faced with the decision of how much to contribute to either help a single identifiable victim or a group of victims, people gave more to the single victim when presented with only one option but their preferences shifted towards the more effective option when presented with both options simultaneously (for related studies on the role of emotions on donation behavior see Small, Loewenstein, & Slovic, 2007; Dickert, Sagara, & Slovic, 2011). In contrast to Hsee’s study, this paradigm did not feature two attributes with different degrees of evaluability. But these results still suggest that many people do share the broadly consequentialist aim of helping as many individuals as possible but, in some contexts, find it hard to evaluate relevant options in light of this goal. It is possible that the evaluability bias might also be driving submaximizing preferences in other contexts where the consequentialist dimension of available options is too difficult to evaluate.

Put together, this prior research suggests that the evaluability bias can influence decision-making both generally and, more specifically, in the context of altruistic decisions. However, this bias has not yet been demonstrated in the context of charitable donation, and in particular with respect to the weight people give to overhead ratio as opposed to cost-effectiveness. Other unsolved questions are the factors that could be responsible for people’s preoccupation with overhead ratio, the extent to which the evaluability bias might be generalizable to other domains, and which mechanisms underlie this bias. In the following, we present four studies that aim to address these questions.

## 3 Study 1

In this study, we aimed to empirically investigate the evaluability bias in the context of charitable donations. If the hypothesis of the evaluability bias is correct, we can expect that people donate on average more to charities with better measures on those attributes that have high evaluability. We assume that most people perceive overhead ratio as an attribute that is easier to evaluate than cost-effectiveness, given most people lack knowledge about the cost-effectiveness of charities in general and thus lack a reference point, which is needed to usefully evaluate this attribute.

As already shown by Hsee (1996), the evaluability of attributes that are difficult to evaluate in separate evaluation can be increased when people are able to evaluate two options jointly. This gives people a reference point to compare the measures. We therefore hypothesized that when people are able to evaluate two charities jointly, the evaluability of cost-effectiveness should increase and people will put more weight on cost-effectiveness compared to overhead ratio. Thus, we expected a preference reversal between overhead ratio and cost-effectiveness. We predicted that in joint-evaluation donations would be higher to the more cost-effective (from now on referred to as *effective*) charities, while in separate-evaluation donations would be higher to the charities with lower overhead ratios.

### 3.1 Method

Ninety-four US American subjects (32 female) were recruited through Amazon’s Mechanical Turk (Buhrmester, Kwang, & Gosling, 2011), with a mean age of 29 years (*SD* = 9.16). Subjects completed the study online and received $0.50 in return for their participation.^3^

Subjects were randomly assigned to one of three groups in equal numbers. In the *joint-evaluation* condition, subjects were informed about the attributes of two charities (Charities A and B), while in the two *separate-evaluation* conditions subjects were presented with only one charity: either Charity A or Charity B. No further information about the nature or aims of these charities was presented. As such, the only difference between the charities was their cost-effectiveness and overhead ratio. In the joint-evaluation condition people were presented with the two hypothetical charities depicted in Table 1. The details of the two attributes were presented in a sentence the following way (example given for charity A): “Per $1,000 donated, $600 go into administration. With the remaining $400, 5 lives are saved.”

Both charities featured the two attributes administrative costs (overhead ratio) and saved lives (cost-effectiveness). While Charity B had a lower overhead ratio than Charity A, Charity A was more than twice as effective in terms of how many lives it saved per input dollar.

After being presented with this information, subjects were asked to indicate how much they hypothetically would be willing to donate (from now on referred to as *donate*) on a scale from $100 to $500. Subjects in the joint-evaluation group were able to indicate this for both charities separately (e.g., $0 to one charity and $350 to the other), but they were not allowed to give more than $500 in total.

We decided that the minimum donation amount should not be zero in order to make the design more similar to Hsee’s music dictionary study (1996) in which subjects were asked to indicate their willingness-to-pay between $10 and $50. However, during analysis we observed that several subjects still donated $0 despite our instruction. For all studies we decided not to exclude subjects from the analysis who donated $0 as this occurred in all conditions. All reported results remain stable when these subjects are excluded.

### 3.2 Results

**Figure 1:**
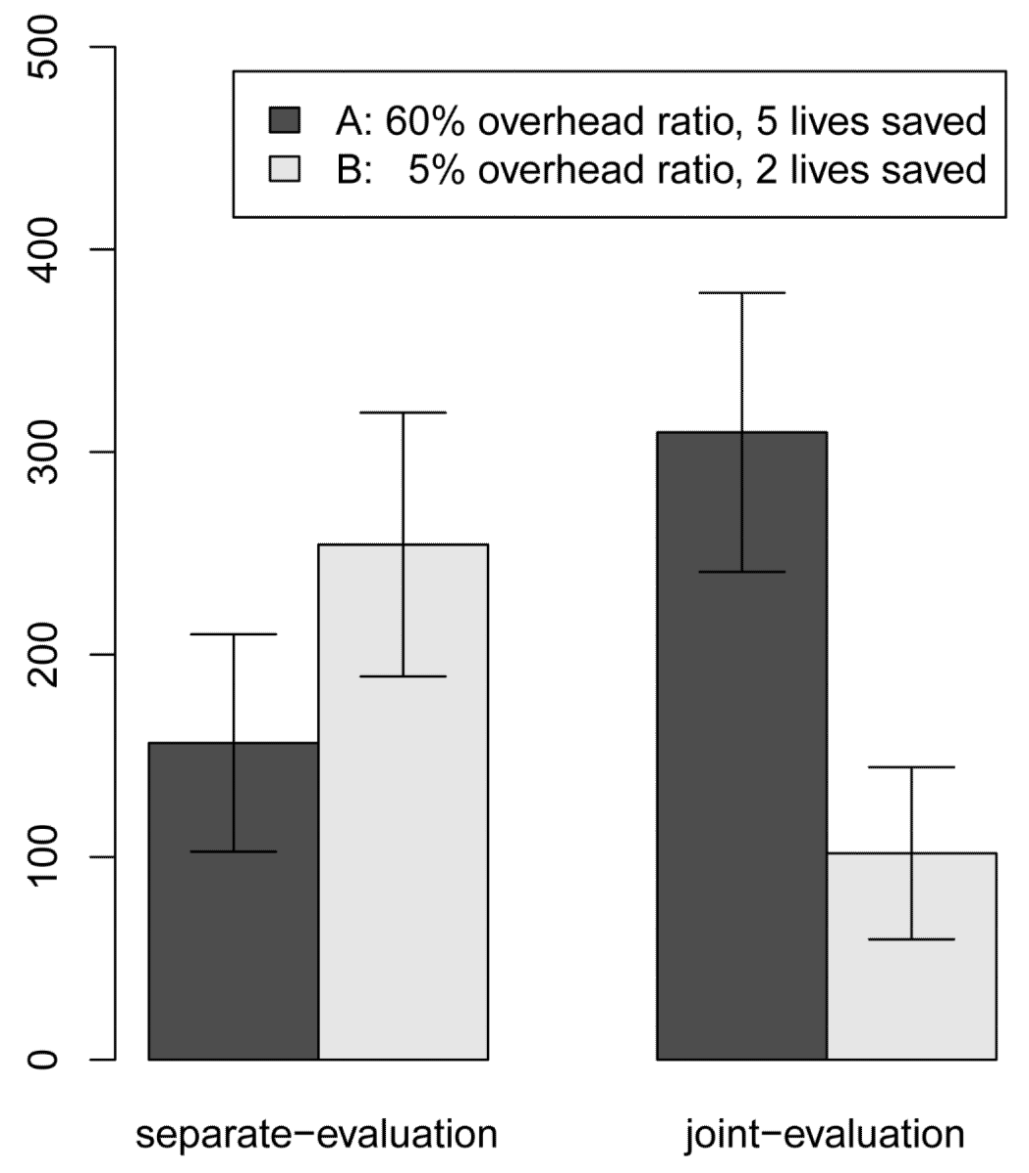
Average donations depending on separate vs. joint-evaluation of charities, Study 1.

The results support our hypothesis that joint-evaluation increases donations to effective charities. In the separate-evaluation conditions, results from an independent samples t-test, *t*(54) = 2.38, p = .021, revealed that on average^4^ subjects donated significantly more money to Charity B (*M* = 254.31, *SD* = 171.13) than to Charity A (*M* = 156.37, *SD* = 141.04). In contrast, subjects in the joint-evaluation condition, who were presented with both charities simultaneously, donated significantly more money to Charity A (*M* = 309.68, *SD* = 191.01) than to Charity B (*M* = 101.87, *SD* = 117.84) as shown by a paired-sample *t* test, *t*(52) = 5.24, p < .001. As such, the predicted a preference reversal^5^ was observed, *t*(86) = 5.74, p < .001 (see Figure 1). Despite the clear preference reversal, considerable individual variance was observed, which can be seen in the rank graphs in the Appendix.

Subjects who were presented with both charities (joint-evaluation) donated larger amounts to the more effective charity (Charity A), while subjects who were only presented with one charity (separate-evaluation) donated more money to the one with the lower overhead ratio (Charity B).

### 3.3 Discussion

In this study, we investigated people’s donation behavior by manipulating the evaluability of cost-effectiveness. We found that joint-evaluation donations are higher to the effective charity whereas separate-evaluation donations are higher to the charity featuring the lower overhead ratio. This suggests that most people primarily value cost-effectiveness but that they are unable to evaluate it in separate-evaluation. People may find it difficult to evaluate charities with respect to their cost-effectiveness when presented with only one charity, without further information serving as a reference point. It is hard to determine how good a charity is that saves X lives per $Y, so people instead appear to focus on the charity’s overhead ratio. The observation that these preferences reverse when direct evaluation of cost-effectiveness is made possible (by comparing multiple charities) suggests that most people nevertheless do value—at least in large part—maximizing positive impact. If people gave decisive weight to overhead ratio, donations to the charity with lower overhead ratio should stay the same in joint-evaluation. However, we found that when people were presented with two charities simultaneously, their choices were guided by cost-effectiveness even in cases where the overhead ratio is high.

## 4 Study 2

The data of the first study suggest that people primarily value cost-effectiveness but find it difficult to evaluate this attribute when they lack a reference point for comparison. That people primarily value cost-effectiveness is therefore consistent with the fact that they nevertheless donate more to charities featuring lower overhead ratios in separate-evaluation. The first study, however, did not fully address several questions about people’s concern with overhead ratio. It could be that people value only cost-effectiveness but (mistakenly) believe that low overhead ratio is a good indicator of high cost-effectiveness and therefore donate more to charities with lower overhead ratio in separate-evaluation. Alternatively, people might see intrinsic value in not “wasting” their money by giving it to the charities’ non-needy staff or to advertisement. They may give weight to the “moral quality” of their act and not only to its impact: A donation where a larger percentage of the money reaches those in need may be perceived as purer and more direct, and thus as morally more attractive, despite having a potentially lower impact. This would imply that people do regard low overhead ratio as valuable for its own sake, even if they ultimately value it less than they value cost-effectiveness (i.e., saving more lives).

In Study 2 we tested these conflicting hypotheses by investigating whether the preference reversal between joint- and separate-evaluation observed in Study 1 would be sensitive to differences in overhead ratio. To this end, we replicated the first study with the modification of presenting a range of charities that vary in their degree of overhead ratio. We argue that, in case people value low overhead ratio for its own sake, donations to charities with low overhead ratio will be higher even in joint-evaluation. However, if people do not value low overhead ratio for its own sake but merely as an indicator of high cost-effectiveness, donations should not be influenced by the overhead ratio in joint-evaluation.

### 4.1 Method

Two hundred and one US American subjects (83 female) were recruited through Amazon’s Mechanical Turk, with a mean age of 32.24 years (*SD* = 10.87). Subjects received $0.45 in return for their participation.

Subjects were equally distributed and randomly assigned to one of seven groups in which they were presented to either one (*separate-evaluation*) or two (*joint-evaluation*) charities. In total the study included four different charities (Table 2). Three of them featured relatively high cost-effectiveness (5 lives saved) and varying degrees of overhead ratios from low (5%; *Charity A*), medium (30%; *Charity B*), to high (90%; *Charity C*). The fourth charity featured a low overhead ratio (5%) and low cost-effectiveness (2 lives saved; *Charity D*). The charities were presented in the same way as in the first study. There were four separate-evaluation conditions (one for each charity), and three joint-evaluation conditions that included the ineffective charity as well as each of the more effective charities.^6^

As in the first study, subjects were asked to indicate how much they would donate.

### 4.2 Results

**Figure 2:**
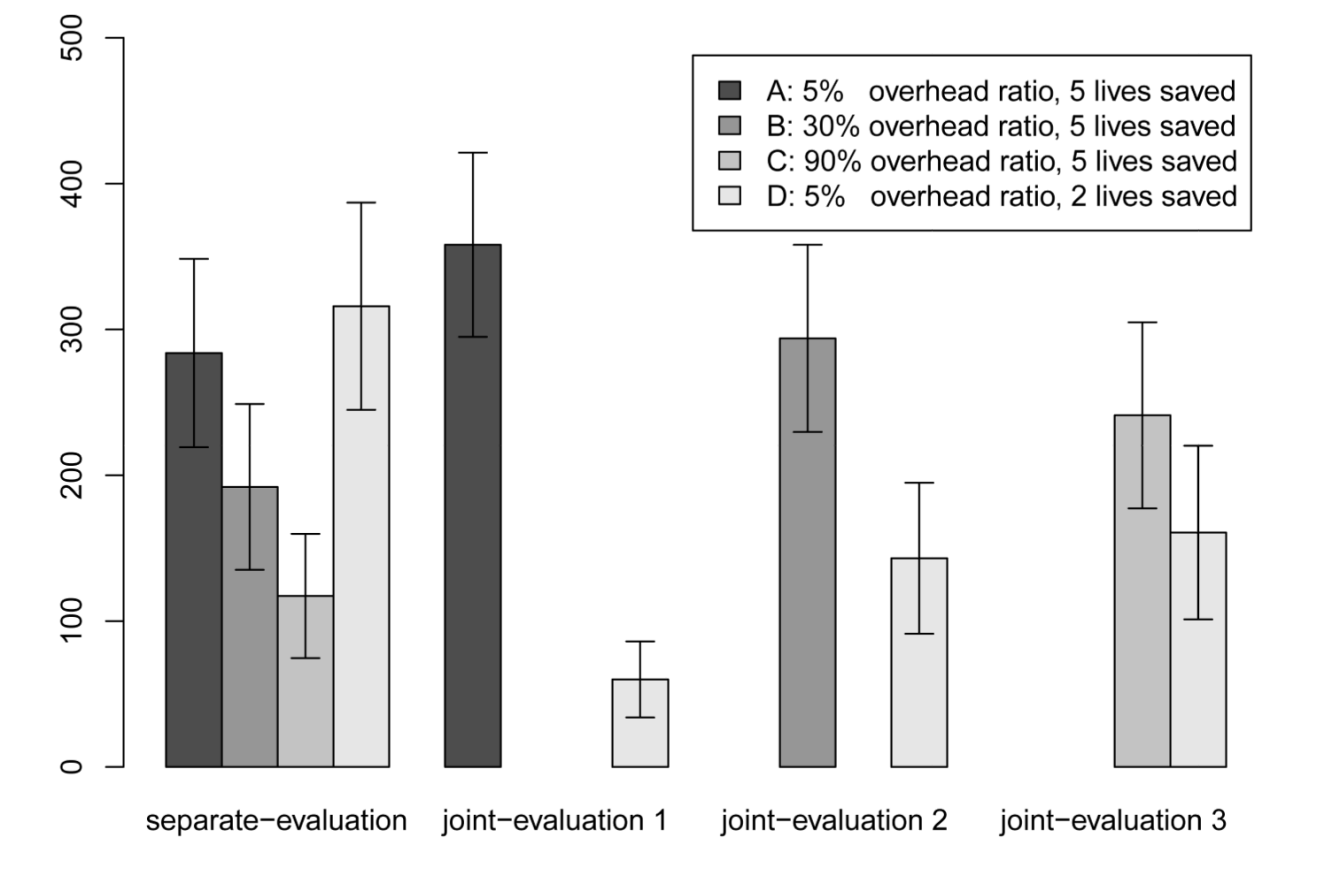
Average donations to charities featuring different overhead ratios, Study 2.

As shown in Figure 2, the data reveal preference reversals in line with the results of Study 1. In the separate-evaluation conditions, donations were higher the lower the overhead ratio was, independently of cost-effectiveness. Subjects on average donated significantly more money to Charity A (*M* = 283.79, *SD* = 169.69) than to Charity B (*M* = 192.00, *SD* = 152.18), *t*(56) = 2.19, p = .033 and significantly more to Charity B than to Charity C (*M* = 117.24, *SD* = 112.02), *t*(53) = 2.15, p = .036. There was no significant difference between donations to the two charities with the same overhead ratio, Charity A and Charity D (*M* = 315.89, *SD* = 183.27), *t*(54) = 0.69, p = .496. In each joint-evaluation condition, however, donations to the effective charity were higher than donations to the ineffective charity. In the first joint-evaluation condition subjects donated more to Charity A (*M* = 358.03, *SD* = 162.91) than to Charity D (*M* = 60.00, *SD* = 67.27), *t*(36) = 8.95, p < .001. In the second joint-evaluation condition subjects donated more to Charity B (*M* = 293.79, *SD* = 168.74) than to Charity D (*M* = 143.10, *SD* = 136.09), *t*(54) = 3.74, p < .001. And in the third joint-evaluation condition subjects donated more to Charity C (*M* = 241.07, *SD* = 164.45) than to Charity D (*M* = 160.71, *SD* = 153.57), *t*(54) = 1.89, p = .064. As such, three preference reversals were observed between Charity A and D, *t*(81) = 5.89, p < .001; between Charity B and D, *t*(83) = 5.02, p < .001; as well as between Charity C and D, *t*(81) = 5.47, p < .001.

To investigate whether the overhead ratio was valued for its own sake, we compared the average amounts donated to the ineffective Charity D across the joint-evaluation conditions. We found that donations were higher the higher the overhead ratio of the respective other charity was: Donations to Charity D were also significantly higher in the third (*M* = 160.71) compared to the first (*M* = 60.00) joint-evaluation condition, t(37) = 3.17, p < .001.

In addition, we compared the average amounts donated to the respective effective charities across the joint-evaluation conditions. We found that donations to Charity A (*M* = 358.03) in the first joint-evaluation condition were significantly higher compared to Charity C (*M* = 241.07) in the third joint-evaluation condition, *t*(54) = 2.67, p < .001. Rank graphs in the Appendix display individual donations in greater detail.

Subjects who were presented with only one charity (separate-evaluation) donated larger amounts to charities with lower overhead ratios. Subjects who were presented with two charities (joint-evaluation 1–3) donated more to the more effective charity. Donations to the more effective charities decreased in joint-evaluation as its overhead ratio increased.

### 4.3 Discussion

The results draw a more detailed picture of people’s preferences across the range of different overhead ratios. First, the results of this study support our assumption that people primarily value cost-effectiveness, as shown in the higher donations to the effective charities in all three joint-evaluation conditions. Second, the results support the proposal that people find it difficult to evaluate cost-effectiveness in separate-evaluation. This is shown in the preference reversals between joint- and separate-evaluation and in particular in the observation that people donate the same amount of money to charities with equal overhead ratios but different cost-effectiveness in separate-evaluation. Third, the fact that in joint-evaluation people donate less to equally effective charities with higher overhead ratios suggests that they value low overhead ratio for its own sake. This is also shown in the observation that donations are higher (although not statistically significantly in all cases) in joint-evaluation to the ineffective charity with low overhead ratio, the higher the overhead ratio of the other charity is. However, our studies cannot rule out the possibility that people take high overhead ratio to indicate something else they disvalue, such as wastefulness or corruption.

The results of studies one and two indicate that people care about both the cost-effectiveness and the overhead ratio of a charity but it remains unclear whether these results are specific to lives saved or also can be applied to other domains. This question, we addressed in a third study.

## 5 Study 3

In a third study, we were interested in whether the effect observed in the first two studies can be applied to charities in other domains. In the previous studies, cost-effectiveness was defined in terms of how many lives are saved per dollar. But in other domains the cost-effectiveness of a charity could be measured in other ways. Notably, some charitable causes might elicit less strong emotional reactions than that of directly saving human lives. In this study we aimed to examine to which extent people value the cost-effectiveness of charities dedicated to environmental issues. Thus, while the charities of the first two studies indicated how many lives they could save, in this study the charities indicated the amount of CO_2_ reduced per dollar.

### 5.1 Method

Eighty-four US subjects (35 female) were recruited through Amazon’s Mechanical Turk, with a mean age of 32.56 years (*SD* = 11.06). Subjects received $0.45 in return for their participation.

The design was identical to the first study with the only difference that this time both charities were environmental charities. Subjects again were either presented with only one charity or with two charities simultaneously. Charity A again featured an overhead ratio of 60% and had a cost-effectiveness of reducing 500 tons of CO_2_ per $1,000, while Charity B featured a low overhead ratio of only 5% while reducing only 200 tons of CO_2_ per $1,000.

As in the first study, subjects were randomly assigned to one of the three groups (joint-evaluation or separate-evaluation A or B) and were asked to indicate how much they would donate.

### 5.2 Results

**Figure 3:**
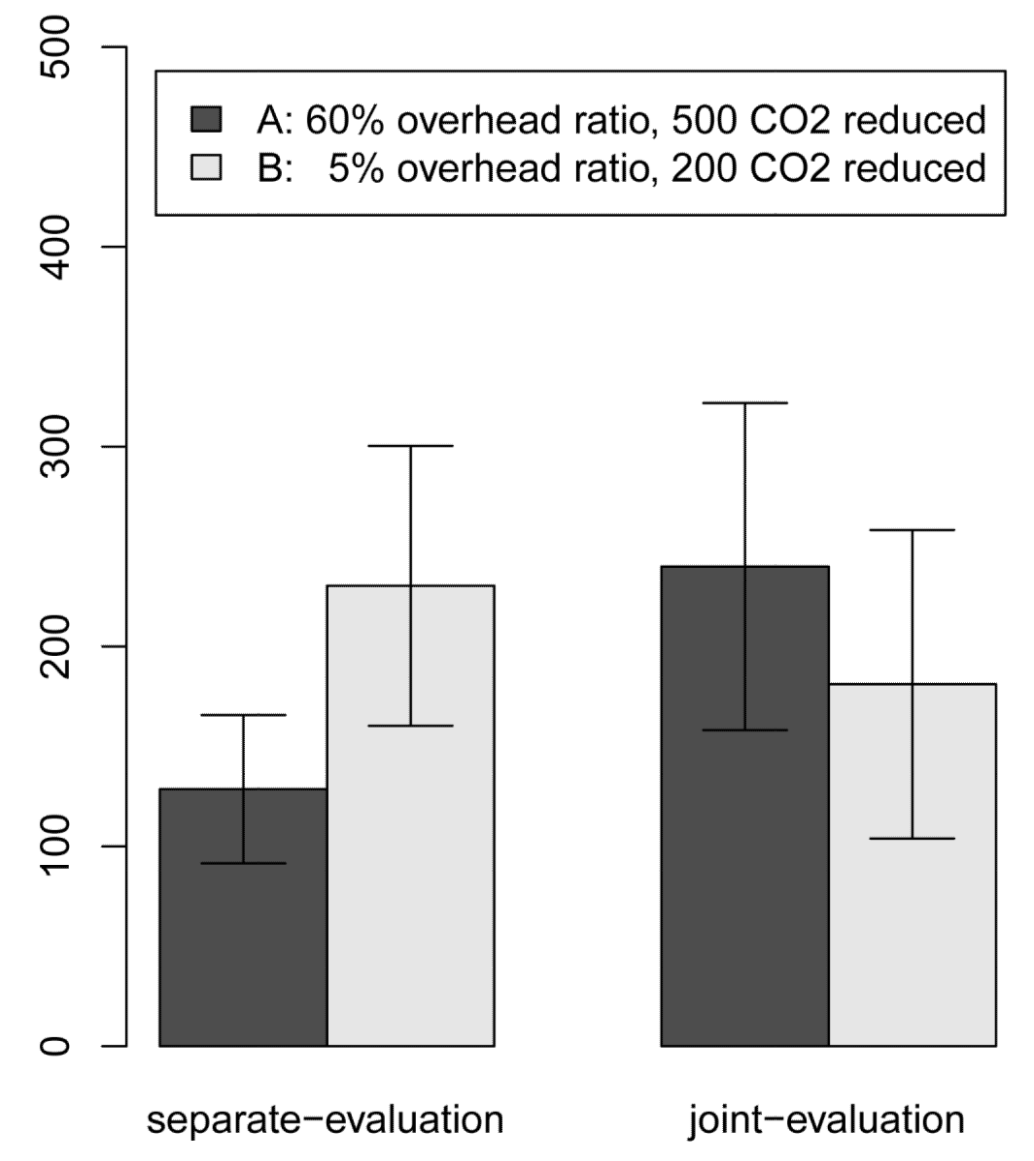
Average donations to environmental charities, Study 3.

In the separate-evaluation condition, subjects donated on average significantly more to Charity A (*M* = 128.57, *SD* = 95.67) than to Charity B (*M* = 230.36, *SD* = 180.58), *t*(41) = 2.64, p = .012. In the joint-evaluation condition, subjects donated not significantly more to Charity A (*M* = 240.00, *SD* = 206.88) compared to Charity B (*M* = 181.11, *SD* = 195.12), *t*(52) = 1.08, p = .287. Donations to the effective charity were significantly higher in joint-evaluation compared to separate-evaluation condition, *t*(36) = 2.54, p = .01, while the donations to the ineffective charity did not differ between the two conditions, *t*(52) = 0.97, p = .33. Thus, we still observe a preference reversal, *t*(79) = 2.89, p = .004 (see Figure 3). Rank graphs in the Appendix display individual donations in greater detail.

Subjects who were presented with only one environmental charity (separate-evaluation) donated larger amounts to the charity featuring the lower overhead ratio (Charity B), while donations did not differ significantly when subjects were presented with both charities (joint-evaluation).

### 5.3 Discussion

The results of the third study demonstrate that the effect found in the previous studies is found in an environmental context as well as in the context of saving lives. However, in this study the difference in average donations in the joint-evaluation condition was not significant, which might be due to the fact that people don’t see a strong difference between reducing between 200 and 500 tons of CO_2_.

In the three studies so far, we were able to consistently demonstrate a preference reversal between cost-effectiveness and overhead ratio, depending on whether charities were evaluated jointly or separately. The results seem to indicate that people primarily value cost-effectiveness while still valuing overhead ratio to a lesser extent. How strongly people value cost-effectiveness depends on the charity’s cause.

## 6 Study 4

In a final study we aimed to shed some light on the question of *why* overhead ratio in separate-evaluation seems easier to evaluate than cost-effectiveness. We reason that this is the case because overhead ratio is usually presented in form of an actual ratio, while cost-effectiveness is presented in form of an open-ended scale—a situation we tried to model in our studies one to three. People might assume that ratios are usually set up in such a way that 0% represents the lowest practically achievable value and 100% the highest, while all options in between are roughly equally distributed. In cases where these assumptions are empirically given, people have reference points available that allow them to assess how good or bad the option in question is. Thus, compared to open-ended scales that don’t have any inherent reference point, ratios are easier to evaluate.

To test the hypothesis that the representational form of the attribute changes its evaluability, we tried to increase the evaluability of cost-effectiveness by presenting it in form of a ratio as well. We predicted that donations to an ineffective charity would be lower in separate-evaluation when cost-effectiveness is presented as a ratio, because a presentation as a ratio should make it clear that the charity described is ineffective.

### 6.1 Method

One hundred and forty-three US American subjects (48 female) were recruited through Amazon’s Mechanical Turk, with a mean age of 30.76 years (*SD* = 9.98). Subjects received $0.3 in return for their participation.

The design was between-subjects and consisted of two conditions, which presented the statistics of a charity analogous to the previous studies. The charity’s overhead ratio (5%) and cost-effectiveness number (2 lives saved) were kept constant in both conditions. However, cost-effectiveness was either presented in form of a ratio or not in form of a ratio. In the non-ratio condition it was stated that “2 lives are saved” while for the ratio condition it was stated that “2 lives out of every 10 who are at risk are saved”. Presenting the attribute in form of a ratio should increase the evaluability of cost-effectiveness in separate-evaluation because it will give people a rough reference point: Saving 2 lives out of 10 appears worse compared to saving 2 lives without having a reference point.

### 6.2 Results

**Figure 4:**
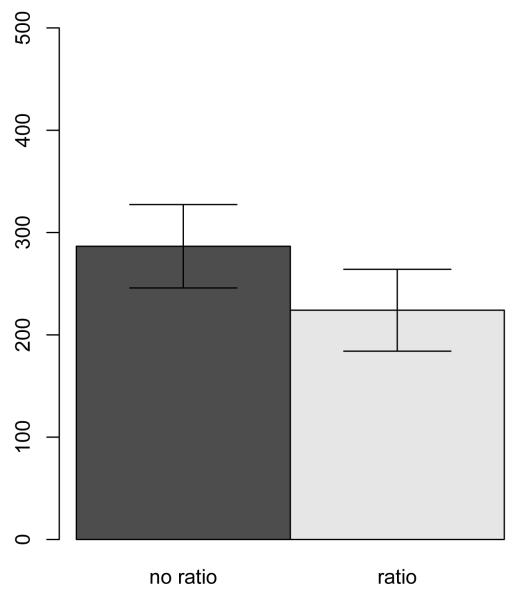
Average donations to charities presenting cost-effectiveness in form of a ratio or not, Study 4.

As shown in Figure 4, subjects donate significantly more to the charity when its cost-effectiveness was not presented in form of a ratio (*M* = 286.59, *SD* = 169.58) compared to when it was presented in form of a ratio (*M* = 224.07, *SD* = 161.48), *t*(132) = 2.19, p = .031. A rank graph in the Appendix displays individual donations in greater detail

Subjects who saw the charity’s cost-effectiveness in form of a ratio (saving 2 lives out of 10 who are at risk) donated on average smaller amounts than subjects who saw the cost-effectiveness not in form of a ratio (saving 2 lives).

### 6.3 Discussion

The results of study four support our suggestion that presenting cost-effectiveness in form of a ratio makes it easier for people to evaluate it in separate-evaluation. Presumably, when cost-effectiveness is presented in form of a ratio, people have a reference point available, which helps them to evaluate the attribute. As such, when presented with a charity that saves 2 lives, people evaluate the charity positively because saving 2 lives seems like a good thing. On the other hand, a charity that saves only 2 lives out of 10 who are at risk is evaluated less positively because people are now able to compare the number of saved lives with the number of unsaved lives. The lower this ratio (20% in this case), the more negatively people are going to evaluate the charity. These considerations seem to suggest one mechanism that may be responsible for an attribute’s evaluability: the inherent reference point of a ratio number increases its evaluability. This may partly explain people’s preoccupation with overhead ratio in the context of charity.

Note that our study has shown only that by presenting cost-effectiveness in an unfavorable ratio donations can be lowered. It remains open whether this technique could be applied to increase donations as well. Thus, further research is needed until it can be decided whether it should be suggested to charities to present their cost-effectiveness in form of a ratio.

The observation that people make use of the inherent reference point of a ratio is in line with other findings (Baron, 1997; Fetherstonhaugh, Slovic, Johnson, & Friedrich, 1997; Slovic, 2007). Fetherstonhaugh et al. (1997), for example, showed that people believed saving a fixed number of lives is more beneficial if the total amount of people at risk is smaller, in which case the ratio of saved people appears higher (for a related study see Erlandsson, Björklund, & Bäckström, 2013). Kleber, Dickert, Peters, & Florack (2013) found that people who are more numerate tend to donate more to charities with higher the higher helping ratio than people who are less numerate.

The results of this study suggest one mechanism that may be responsible for an attribute’s evaluability: the inherent reference point of a ratio number increases its evaluability. This may partly explain people’s preoccupation with overhead ratio in the context of charity.

## 7 General discussion

Research shows that people attribute a puzzling and disproportionate degree of importance to charities’ overhead ratio (administrative expenses), even though overhead ratio is a poor predictor of cost-effectiveness and, with that, how much good can be done per dollar donated. In this article, we have provided evidence that many people prefer low overhead ratio to high cost effectiveness partly because the former is easier to evaluate. When being given the possibility to draw an appropriate comparison, many people’s preferences reverse, showing that people in fact primarily care about cost-effectiveness rather than overhead ratio. This phenomenon has potential practical implications that we highlight below. It is also, we have proposed, just one instance of the more general “evaluability bias”—the tendency to weight the importance of an attribute in proportion to its ease of evaluation.

Over four studies, we have presented evidence supporting the following conclusions:

First, people appear to primarily value cost-effectiveness when choosing how much to donate to a charity, but they give less weight to cost-effectiveness when its evaluability is low compared to overhead ratio. A robust finding throughout our studies is that, when people evaluate charities jointly, average donations to the more effective charity are higher, whereas these preferences can reverse in separate-evaluation. This seems to be the case in the context of directly saving lives and reducing CO_2_. We suggest that this is due to the fact that people have a readily available reference point for evaluation when they are able to compare two charities, which allows them to judge how good or bad the presented charity is with respect to its cost-effectiveness, i.e., the evaluability of cost-effectiveness is higher in joint-evaluation compared to separate-evaluation.

Second, most people value a low overhead ratio even if this means that a given donation has less overall impact. But as soon as the evaluability of cost-effectiveness increases donations shift to more effective charities, indicating that most people primarily value cost-effectiveness.

Third, our fourth study suggests that the higher relative evaluability of overhead costs is, at least in part, due to their being typically presented in the form of a ratio, which, we have argued, is associated with an inherent reference point. This study thus has a potential practical lesson: presenting cost-effectiveness in the form of a ratio number can increase its evaluability and thus increase the relative weight many people assign to it.

Taken together, these four studies indicate that the evaluability bias has a large effect on decisions about charitable giving. However, our studies do not demonstrate that people err in expressing their own values. If people really valued low overhead ratio for its own sake, giving more relative weight to overhead ratio can be considered as a reasonable heuristic in the absence of a clear evaluation of cost-effectiveness (as in the separate-evaluation conditions in our studies). However, people are very likely not applying this heuristic deliberately, so that it is often used inappropriately, in which case it is justified to call it a cognitive bias. For example, if people really valued charities’ cost-effectiveness, there should be a much higher general interest in the scientific evaluation of charities’ cost-effectiveness in real life. But that’s not what we observe (Goggins Gregory & Howard, 2009). In contrast, many people seem to be preoccupied with charities overhead ratios (Sellers, 2012), even though—as our studies strongly suggest—people value cost-effectiveness more than overhead ratio. For donors in real life, it does not take much effort to find the most cost-effective charities: a website visit to cost-effectiveness evaluators, such as *GiveWell* or *Giving What We Can*, would be sufficient to find cost-effectiveness rankings of charities and extensive scientific reviews.

Our studies did not fully address the question of why people care about low overhead ratio. For example, it might be that people don’t realize that a charity, like any for-profit organization, requires administrative expenses to optimize the organization and ensure its impact. Furthermore, people might not disvalue high overhead ratio per se, but only when money is being wasted, i.e., not used optimally to maximize cost-effectiveness.

The study designs differ from real life situations where people are considering donating to charity. First, the subjects’ judgments were hypothetical and real donation behavior might be different. However, hypothetical judgments indicate relevant *attitudes* towards charitable giving. In addition, research has shown that such decisions in hypothetical situations are generalizable to real life (Ariely, 2008). Second, in the present studies, cost-effectiveness was clearly indicated, which usually is not the case in real life situations. In situations where the numbers are not given, the evaluability of cost-effectiveness is decreased further.

If people chose charities to donate to more wisely, many thousands of individuals in need could be helped more effectively (Ord, 2012), and possibly institutional donors (including government) could benefit too. As our studies suggests, potential donors appear to be motivated to maximize the impact of their donation. There is an urgent need for finding ways to help donors make better decisions in order to shift resources towards cost-effective organizations. One practical implication of this result is the need to better inform people about the irrelevance of overhead ratio, as already pioneered by the campaign *The Overhead Myth*.^7^ Another way to help people overcome the evaluability bias is to change their decisional environment (Thaler & Sunstein, 2009) in a way that increases the evaluability of certain attributes, e.g., charity recommending websites could allow for explicit comparison of cost-effectiveness between charities. As suggested by the present research, this can increase the role of consequentialist, cost-benefit analysis in decision-making.

## Figures and Tables

**Table 1:** Charities presented in Study 1.

	Charity A	Charity B
Administrative costs	$600	$50
Saved lives	5	2

**Table 2:** Charities presented in Study 2.

	Charity A	Charity B	Charity C	Charity D
Administrative costs	$50	$300	$900	$50
Saved lives	5	5	5	2

Note: As in Study 1, the details of the two attributes were presented in following way: “Per $1,000 donated, $50 go into administration” and “With the remaining $950, 5 lives are saved” for Charity A.

## Footnotes

1 http://givewell.org.

2 http://givingwhatwecan.org.

3 To ensure the quality of the data, subjects who did not pass an attention check question were excluded from the data analysis (Aust, Diedenhofen, Ullrich, & Musch, 2013). The attention check constituted an item at the end of the study where subjects were told to ignore a certain question and instead write “I read the instructions”. The numbers of subjects who passed/failed the attention check were 94/11, 201/41, 84/13, and 143/13 for the four studies, respectively.

4 For simplicity, we will omit “on average” henceforth.

5 According to the formula to assess preference reversals by Hsee (1996): $t=\frac{\left(M_{JA}-M_{JB})-(M_{SA}-M_{SB}\right)}{{\left(S_{J}^{2}∕N_{J}+S_{SA}^{2}∕N_{SA}+S_{SB}^{2}∕N_{SB}\right)}^{1∕2}}$ where: *M*_*JA*_, *M*_*JB*_, *M*_*SA*_ and *M*_*SB*_ are the respective means for joint and separate presentation of options *A* and *B*; $S_{J}^{2}$, $S_{SA}^{2}$ and $S_{SB}^{2}$ are the respective variances; and $N_{J}^{2}$, $N_{SA}^{2}$ and $N_{SB}^{2}$ are the respective numbers of subjects.

6 For exploratory reasons, study two and three contained a short questionnaire at the very end of the study about general donation behavior and attitudes toward charity (e.g., amount donated last year, attitudes toward overhead ratio and cost-effectiveness). There were no significant differences and correlations found.

7 http://overheadmyth.org.

## Appendix 1

The following rank graphs display the relative ranks of all subjects’ donations in all studies. Despite individual variance, in joint-evaluation average donations are higher to the effective and in separate-evaluation to the ineffective charity.

**Study one:**

**Study two:**

**Study three:**

**Study four:**

## References

[R1] Ariely D (2008). Predictably irrational. The Hidden Forces That Shape Our Decisions.

[R2] Aust F, Diedenhofen B, Ullrich S, Musch J (2013). Seriousness checks are useful to improve data validity in online research. Behavior Research Methods.

[R3] Baron J (1997). Confusion of relative and absolute risk in valuation. Journal of Risk and Uncertainty.

[R4] Baron J (2005). Thinking and deciding.

[R5] Baron J, Szymanska E, Oppenheiemer DM, Olivola CY (2011). Heuristics and biases in charity. The science of giving: Experimental approaches to the study of charity.

[R6] Bazerman MH, Loewenstein GF, White SB (1992). Reversals of preference in allocation decisions - judging an alternative versus choosing among alternatives. Administrative Science Quarterly.

[R7] Bedsworth W, Gregory AG, Howard D (2008). The Bridgespan Group.

[R8] Buhrmester M, Kwang T, Gosling SD (2011). Amazon’s Mechanical Turk: A new source of inexpensive, yet high-quality, data?. Perspectives on Psychological Science.

[R9] CARE (2014). CARE USA Financial Information.

[R10] Dickert S, Sagara N, Slovic P (2011). Affective motivations to help others: A two-stage model of donation decisions. Journal of Behavioral Decision Making.

[R11] Erlandsson A, Björklund F, Bäckström M (2014). Perceived utility (not sympathy) mediates the proportion dominance effect in helping decisions. Journal of Behavioral Decision Making.

[R12] Fetherstonhaugh D, Slovic P, Johnson SM, Friedrich J (1997). Insensitivity to the value of human life: A study of psychophysical numbing. The psychology of peacekeeping.

[R13] Goggins Gregory A, Howard D (2009). The nonprofit starvation cycle. Stanford Social Innovation Review.

[R14] Hsee C (1996). The evaluability hypothesis: An explanation for preference reversals between joint and separate evaluations of alternatives. Organizational Behavior and Human Decision Processes.

[R15] Hsee C (1998). Less is better: When low-value options are valued more highly than high-value options. Journal of Behavioral Decision Making.

[R16] Hsee C, Zhang J (2010). General evaluability theory. Perspectives on Psychological Science.

[R17] Jamison DT (2006). Disease control priorities in developing countries.

[R18] Kahneman D, Kahneman D, Tversky A (2000). Evaluation by moments: past and future. Choices, values and frames.

[R19] Karnofsky H (2007). Which of these boasts is not like the others?.

[R20] Kogut T, Ritov I (2005). The singularity effect of identified victims in separate and joint evaluations. Organizational Behavior and Human Decision Processes.

[R21] Kleber J, Dickert S, Peters E, Florack A (2013). Same numbers, different meanings: How numeracy influences the importance of numbers for pro-social behavior. Journal of Experimental Social Psychology.

[R22] Ord T (2012). The moral imperative toward cost-effectiveness in global health. Center for Global Development.

[R23] Sellers R (2012). Where’d my money go?. Grey Matter Research.

[R24] Singer P (1979). Practical ethics.

[R25] Slovic P (2007). “If I look at the mass I will never act”: Psychic numbing and genocide. Judgment and Decision Making.

[R26] Slovic P, Finucane M, Peters E, MacGregor DG (2002). Rational actors or rational fools: Implications of the affect heuristic for behavioral economics. The Journal of Socio-Economics.

[R27] Small DA, Loewenstein G, Slovic P (2007). Sympathy and callousness: The impact of deliberative thought on donations to identifiable and statistical victims. Organizational Behavior and Human Decision Processes.

[R28] Steinberg RM, Debra. (2010). Ratio discrimination in charity fundraising: the inappropriate use of cost ratios has harmful side-effects. Voluntary Sector Review.

[R29] Thaler RH, Sunstein CR (2009). Nudge: improving decisions about health, wealth, and happiness.

[R30] Wing K, Hager MA (2004). Getting what we pay for: Low overhead limits nonprofit effectiveness. Nonprofit Overhead Cost Project.

